# Association between horizontal violence and turnover intention in nurses: A systematic review and meta-analysis

**DOI:** 10.3389/fpubh.2022.964629

**Published:** 2022-10-06

**Authors:** Yue Zhang, Rulan Yin, Jing Lu, Jianzheng Cai, Haifang Wang, Xiaoqing Shi, Lifen Mao

**Affiliations:** ^1^Department of Nursing, The First Affiliated Hospital of Soochow University, Suzhou, China; ^2^School of Nursing, Medical College of Soochow University, Suzhou, China; ^3^Department of Rheumatology, The First Affiliated Hospital of Soochow University, Suzhou, China; ^4^Faculty of Nursing, Chiang Mai University, Chiang Mai, Thailand

**Keywords:** horizontal violence, turnover intention, nurses, occupational health, meta-analysis

## Abstract

**Background:**

Horizontal violence is common in nurses. Most published studies have focused on horizontal violence and higher turnover rates in nurses; however, it lacks systematic reviews and meta-analyses. The purpose of this review is to quantitatively assess the correlation between horizontal violence and turnover intention in nurses.

**Methods:**

Systematic review and meta-analysis were performed in accordance with PRISMA guidelines. The relationship between horizontal violence and turnover intention in nurses was obtained by systematically searching related literature in four English databases (Cochrane, PubMed, Embase, and CINAHL) and three Chinese databases (SinoMed, CNKI, and Wanfang) (up to 6 March 2022). The relationship between horizontal violence and turnover intention was evaluated using Fisher's *z-*value, which was then converted to *r*. STATA 16.0 was used to perform statistical analysis. The random-effects model was performed to synthesize data.

**Results:**

A total of 14 studies with 6,472 nurses were included. A low-positive correlation of horizontal violence with turnover intention was found (pooled *r*=0.32 [0.29–0.34]). Subgroup analysis showed that sample size and quality were not the source of heterogeneity. Measurement tool was the source of heterogeneity. Although geographic region might not be the source of heterogeneity, further subgroup analysis of the country reveals heterogeneity. The funnel plot and Egger's test showed no publication bias.

**Conclusion:**

Horizontal violence had a low positive correlation with turnover intention in nurses. Nurses who experienced horizontal violence were more likely to leave or change careers than those who did not experience horizontal violence. This finding helps to draw attention to horizontal violence by nursing managers and implement effective interventions for nurses, so as to reduce nurses' turnover.

## Introduction

Horizontal violence (HV), which belongs to internal workplace violence, refers to inter-group conflict, manifested by sabotage, infighting, scapegoating, criticism, and other explicit and implicit non-physical hostilities (1). In the current study, other terms are also used to describe negative behaviors among peers, such as workplace violence, bullying, or workplace incivility (2–4). In recent years, some scholars have made conceptual distinctions between different terms (5). This review focused on the negative behaviors among peers of the same status, without considering the temporal and behavioral differences of negative behaviors. Therefore, horizontal violence is used in this study, and other terms are considered to be forms of horizontal violence. Horizontal violence has many negative effects on nurses. It not only causes negative emotions in nurses (6) and physical symptoms, such as headache and insomnia (7, 8), but also affects the atmosphere of nursing organizations (9, 10) and the quality of nursing work (11). Nurses who have been exposed to horizontal violence for a long time may have the leave intention (12). Currently, sufficient evidence showed that turnover intention is the direct premise of turnover behavior (13). As a high turnover rate has many adverse effects on nurses' own development, nursing quality, patient outcomes, medical organization stability, and other aspects, it has been a wide concern among scholars and nursing managers (14). In conclusion, turnover intention, as an adverse outcome of horizontal violence, is a huge obstacle in nursing career development, and it is necessary to think highly of the association between the two aspects.

Turnover intention, which refers to the tendency of employees to leave their current job and seek other job opportunities, is a key predictor of turnover although it does not necessarily lead to actual turnover (15). Frequent employee turnover reduces organizational efficiency, may cause emotional instability and slack behavior of other employees in the organization, and increases the hospital's investment in nurse training (16). In the case of a shortage of nurses, finding the factors that affect nurses' turnover intention and reducing nurses' turnover intention are the main issues that researchers need to consider.

Current research showed that the reasons for nurses' resignation include salary, negative working environment, excessive workload, and inconsistency with personal expectations (17, 18). However, it is difficult to substantially improve nurses' salaries, reduce their workload and change their social status in the short term. Scholars mostly put forward strategies to reduce nurses' turnover intention from the perspective of improving the organizational atmosphere and providing a healthy working environment (18). In the context of workplace violence, scholars pay more attention to the high prevalence of violence between nurses and patients and pay less attention to the negative behavior between colleagues. As a result, there are still some nurses who lack awareness of horizontal violence and lack the ability to effectively deal with it, so they choose negative coping methods, such as silence and compromise. When it exceeds the tolerance of nurses, they will have the intention to leave or change careers. Bambi et al. (19) showed that in 87.4% of nurses exposed to horizontal violence, as many as 75% showed physical and psychological symptoms, and about 10% showed symptoms of post-traumatic stress disorder. Some junior nurses chose to leave or even change careers due to horizontal violence. Other studies showed that inter-nurse horizontal violence is one of the most destructive problems affecting nursing career development, and nurses often deal with horizontal violence in negative ways such as resignation, retaliation, avoidance, and turnover intention (12). A study occurred in China showed that the prevalence of horizontal violence among nurses is about 56.6%, among which 21.67% are nurses with turnover intention and 33.33% are nurses with change careers intention (20). A multi-center study from the USA showed that 43–46% of participants indicated that horizontal violence did not impact their intention to leave, but 11–16% felt horizontal violence impacted their intention (21).

In conclusion, most studies indicate that horizontal violence is associated with turnover intention, but the proportion of turnover intention varies among studies, and the degree of correlation between horizontal violence and turnover intention is unclear. To date, no meta-analysis has been published on the association between horizontal violence and turnover intention. Therefore, in view of this situation, we conducted a systematic review and meta-analysis to gather the available evidence and more accurately evaluate the correlation between horizontal violence and turnover intention in nurses, so as to provide a recommendation to pay attention to horizontal violence, investigate antecedents, and implement strategies to reduce nurses' turnover intention.

## Materials and methods

### Search strategy

This systematic review and meta-analysis were carried out according to the Preferred Reporting Items for Systemic Review and Meta-Analyses (PRISMA 2020) guidelines (22). A systematic search was performed in the four English databases: Cochrane Library, PubMed, EMBASE, and CINAHL, and three Chinese databases: SinoMed, CNKI, and Wanfang (from inception to March 6, 2022). Keywords used for searching were “horizontal violence” (including “lateral violence”, “horizontal hostility”, “bullying place”, and “workplace incivility”) and nurses, with the retrieval adjusted according to the database, the search strategy is shown in Supplementary File 1. In addition, the list of references in the included articles was searched to obtain additional studies.

### Inclusion criteria

Inclusion criteria were: (1) The participants were nurses, and the violent behavior was from colleagues; (2) investigating the relationship between horizontal violence and turnover intention; (3) reporting data on the correlation between horizontal violence and turnover intention, including Spearman's or Pearson's correlation coefficient (*r*); (4) research design was cross-sectional, case-control, or longitudinal design (using baseline data); and (5) published in English and Chinese.

### Exclusion criteria

Exclusion criteria were: (1) meeting or conference abstracts, case reports, reviews, meta-analysis, letters, pilot studies, qualitative studies, and study protocols; (2) full-text studies not found; (3) duplicate articles and/or data (selected the most recent article); and (4) unclear descriptions of nurse populations and data.

### Data extraction and quality assessment

Two reviewers screened the literature independently according to the inclusion and exclusion criteria. After confirming the included studies, the two authors independently extracted data from each paper, including the first author, year of publication, country, sample size and percent of females, age, working experience, tools of horizontal violence, and Pearson's/Spearman's correlation coefficient (*r*) between horizontal violence and turnover intention. The quality of the included studies was evaluated using the modified Newcastle-Ottawa Scale (M-NOS) (23). There are eight items and a maximum of 10 stars. The higher scores indicate better quality. In this study, ≥5 was defined as low-risk bias and <5 as high-risk bias. Any disagreements between two authors, which cannot be resolved through discussion, should be discussed and adjudicated by the third author.

### Statistical analysis

Stata 16.0 was used for meta-analysis. The random-effects model was used as a synthesizer, as it is more desirable than the fixed-effects model and can provide a wider confidence interval (CI). For correlation coefficients (*r*), Spearman's *r* was first converted to Pearson's *r* (24). Then the pooled estimate of Pearson's *r* by Fisher's exact test *r*-to-*z* transformation was calculated (25). All values were weighted by the reciprocal of the *r* variance, after which the combined *r* of the overall value was converted back for presentation. *I*^2^ was adopted to assess between-study heterogeneity, with thresholds of 25% (low heterogeneity), 50% (moderate heterogeneity), and 75% (high heterogeneity) (26). Subgroup and sensitivity analyses were used to search for sources of heterogeneity. Funnel plots and Egger's test were only combined to assess publication bias when ≥10 studies were included (27, 28), as the power of these tests is too low to distinguish chance from real asymmetry when there are <10 studies (29).

## Results

### Study selection

**Figure 1:**
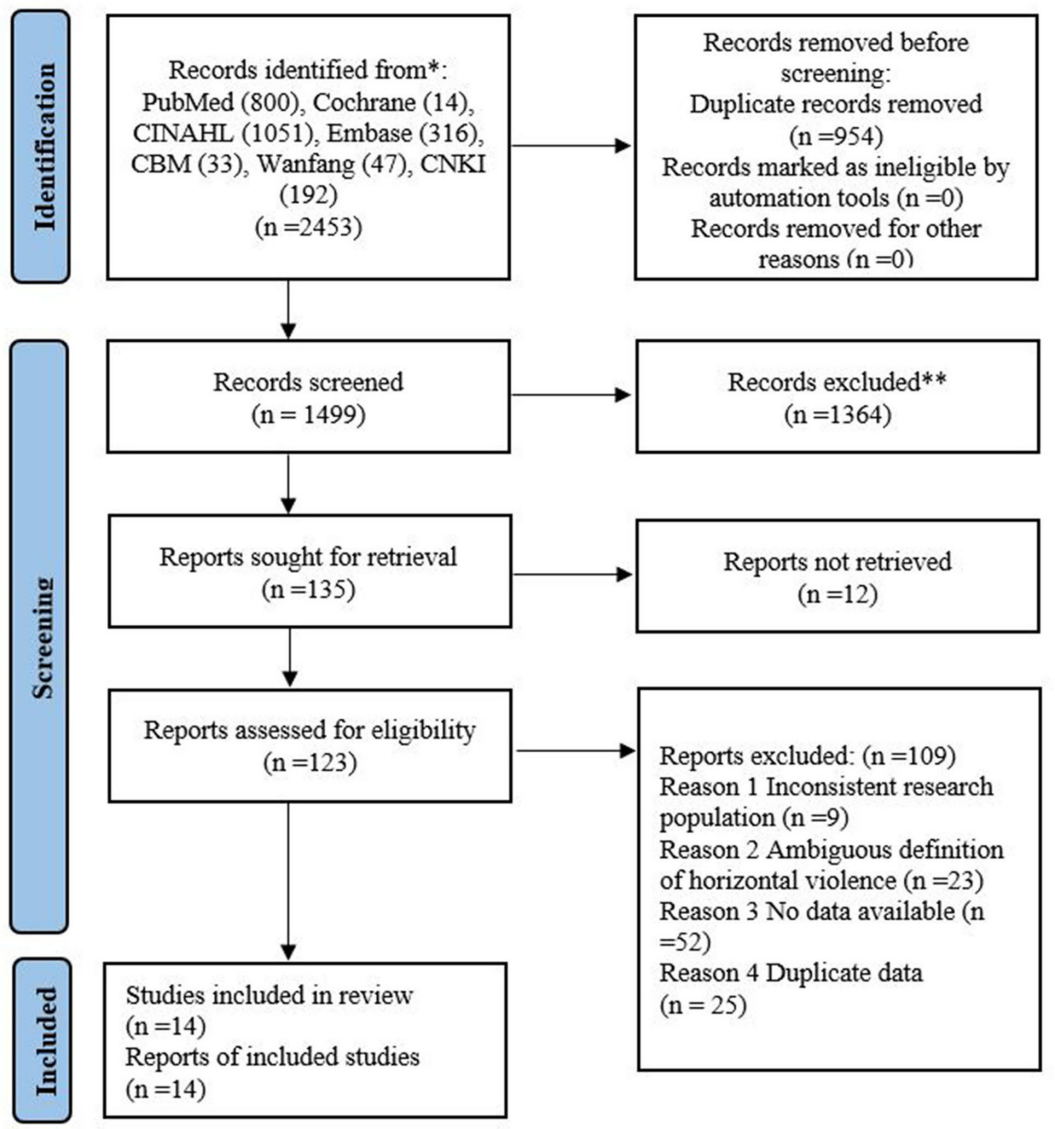
Screening flow chart.

After having assessed the studies by selection criteria, data from 14 studies were included, which involved 6,472 nurses. A flow chart of the study selection process is shown in Figure 1.

### Study characteristics

In this systematic review and meta-analysis, five studies occurred in Canada (4, 30–33), four in America (34–37), two in Korea (38, 39), and one in each of the following countries: China (40), Pakistan (41), and Turkey (42). Ten studies had large sample sizes (≥200 cases) and the others were small sample sizes (<200 cases). NAQ-R scale was most commonly used to measure horizontal violence (five studies), following the WIS scale (three studies). Measuring the quality of studies by M-NOS (0–5 scores), two studies were judged as the high risk of bias (<3 points), and the others were judged as the low risk of bias (≥3 points). Details are shown in Table 1.

**Table 1:** Characteristics of the incorporated studies.

**study**	**Country**	**Sample size**	**Female (%)**	**Age (year)**	**Working Experience (year)**	**Tools of HV**	**r**	**r type**	**Quality**
Simons (34)	US	511	93.3	33.1 ± 9.0	3.7 ± 5	NAQ-R	0.51	Pearson	6
Spence et al. (30)	Canada	612	95	41.3 ± 10.6	11.23 ± 11.27	WIS	0.19	Pearson	5
Leiter et al. (31)	Canada	477	94.8	41.98 ± 10.477	13.55 ± 10.26	WIS	0.19	Pearson	5
Spence et al. (32)	Canada	342	91.5	28.10 ± 6.58	1.04 ± 0.24	NAQ-R	0.32	Pearson	4
Oyeleye et al. (35)	US	61	87	40	11 ± 9.3	the Uncivil Workplace Behaviors questionnaire and WIS	0.496	Pearson	5
Shiau-Ting et al. (40)	China	708	98.2	30.17 ± 7.15	7.17 ± 6.97	NAQ-R	0.389	Pearson	5
Dellasega et al. (36)	US	842	93.5	40.95 ± 15.01	8.32	RAAS	0.24	Spearman	5
Blackstock et al. (4)	Canada	94	85	42	11.8	the 9-item scale from Hutchinson et al.	0.32	Pearson	5
Armmer et al. (37)	US	104	96.2	38.9 ± 10.3	13.7 ± 10.3	BSSQ	0.214	Pearson	4
Munir et al. (41)	Pakistan	668	—	—	—	NAQ-R	0.561	Pearson	5
Yun et al. (38)	Korea	301	98.7	29.23 ± 6.49	6.5 ± 5.72	NAQ-R	0.28	Pearson	6
Arslan et al. (42)	Turkey	574	90.8	36.5 ± 7.6	15.3 ± 8.5	WIS	0.19	Pearson	5
Lee et al. (39)	Korea	170	94.7	28.61	4.02	the Nurses Incivility Scale developed by Guidroz et al.	0.33	Pearson	6
Favaro et al. (33)	Canada	1008	92.5	27.42 ± 6.36	1.18 ± 0.50	NAQ	0.21	Pearson	5
Armmer et al. (37)	US	104	96.2	38.9 ± 10.3	13.7 ± 10.3	BSSQ	0.214	Pearson	4
Munir et al. (41)	Pakistan	668	—	—	—	NAQ-R	0.561	Pearson	5
Yun et al. (38)	Korea	301	98.7	29.23 ± 6.49	6.5 ± 5.72	NAQ-R	0.28	Pearson	6
Arslan et al. (42)	Turkey	574	90.8	36.5 ± 7.6	15.3 ± 8.5	WIS	0.19	Pearson	5
Lee et al. (39)	Korea	170	94.7	28.61	4.02	the Nurses Incivility Scale developed by Guidroz et al.	0.33	Pearson	6
Favaro et al. (33)	Canada	1,008	92.5	27.42 ± 6.36	1.18 ± 0.50	NAQ	0.21	Pearson	5

HV, Horizontal violence; NAQ-R, Negative Acts Questionnaire-Revised; RAAS, The Relational Aggression Assessment Survey; BSSQ, the Briles' Sabotage Savvy Questionnaire; WIS, the Workplace Incivility Scale developed by Cortina et al. (43); NAQ, A shortened (3-item) version of the Negative Acts Questionnaire.

### Correlation between horizontal violence and turnover intention in nurses

**Figure 2:**
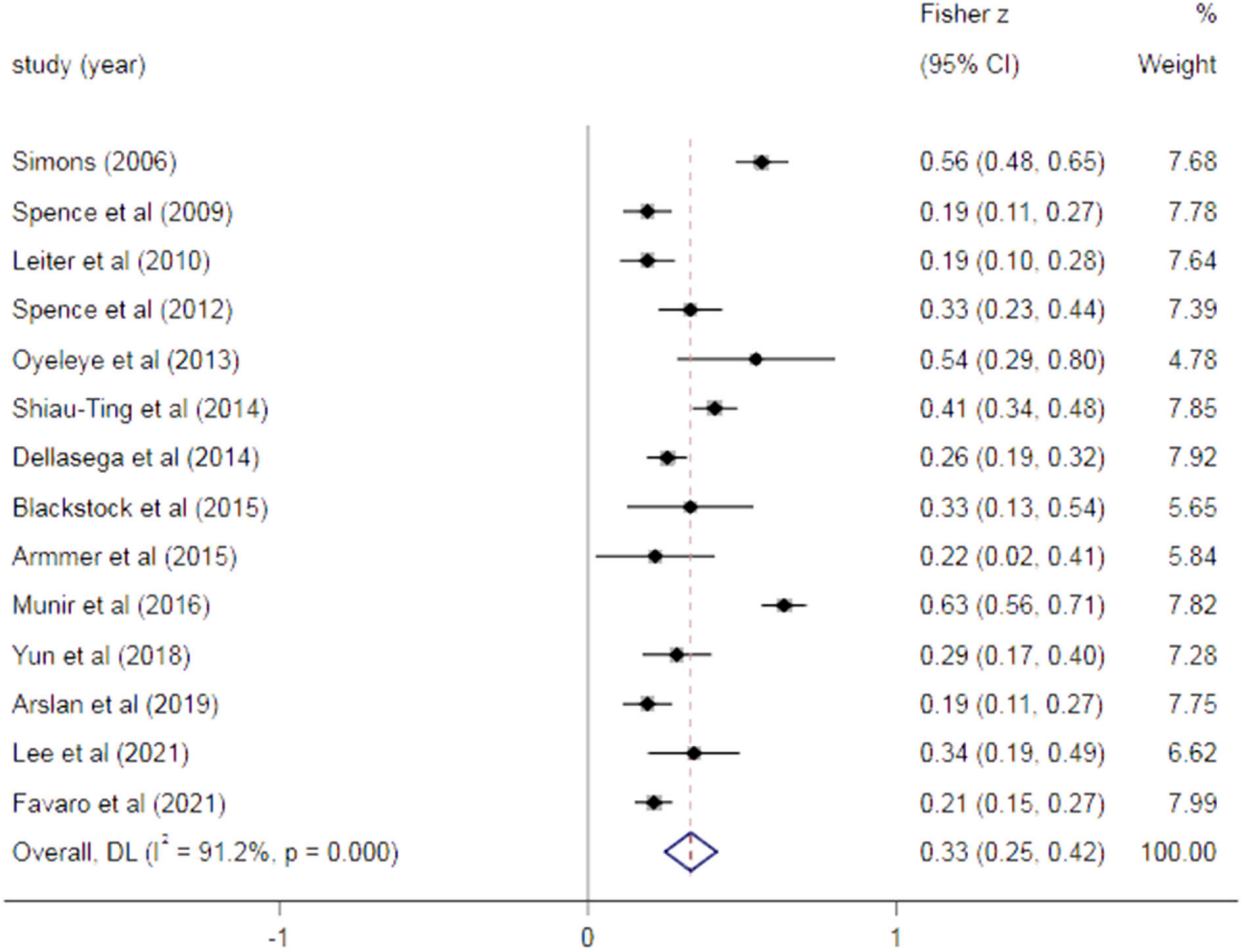
Forest plot of eligible studies.

As shown in Figure 2 14 studies reported a correlation (*r*) between horizontal violence and turnover intention among nurses, and the pooled Fisher *z-*value was 0.33 (95% CI: 0.25–0.42; *I*^2^ = 91.2%, *P* < 0.001). After the *z*-to-*r* back transformation, the pooled *r* was 0.32 (95% CI: 0.29–0.34; *P* < 0.001), and 95% CI (0.24–0.39) does not include the value 0, suggesting a positive relationship between the horizontal violence and turnover intention, and the correlation magnitude is lower.

### Subgroup analysis

Subgroup analysis was conducted based on geographic region, sample size, measurement tools of horizontal violence, and quality. The results showed that measurement tools may be the source of heterogeneity in the meta-analysis of the correlation between horizontal violence and turnover intention. Subgroup analysis showed that the pooled Fisher *z-*value was 0.45 (95%CI: 0.32–0.58) for NAQ-R, which suggests a low-positive relationship. However, WIS and Others were 0.19 (95%CI: 0.14–0.24) and 0.27 (95%CI: 0.21–0.34), respectively, showing that horizontal violence was not associated with turnover intentions. Geographical regions are further divided into subgroups of countries (US, Canada, and Korea), and heterogeneity is found. The results showed that the pooled Fisher *z*-value was 0.39 (0.19, 0.59) in the USA and 0.31 (0.22, 0.40) in Korea, suggesting a low correlation between horizontal violence and turnover intention. In Canada, it was 0.23 (0.18, 0.28), suggesting no correlation (Supplementary File 2).

### Sensitivity analysis and publication bias

**Figure 3:**
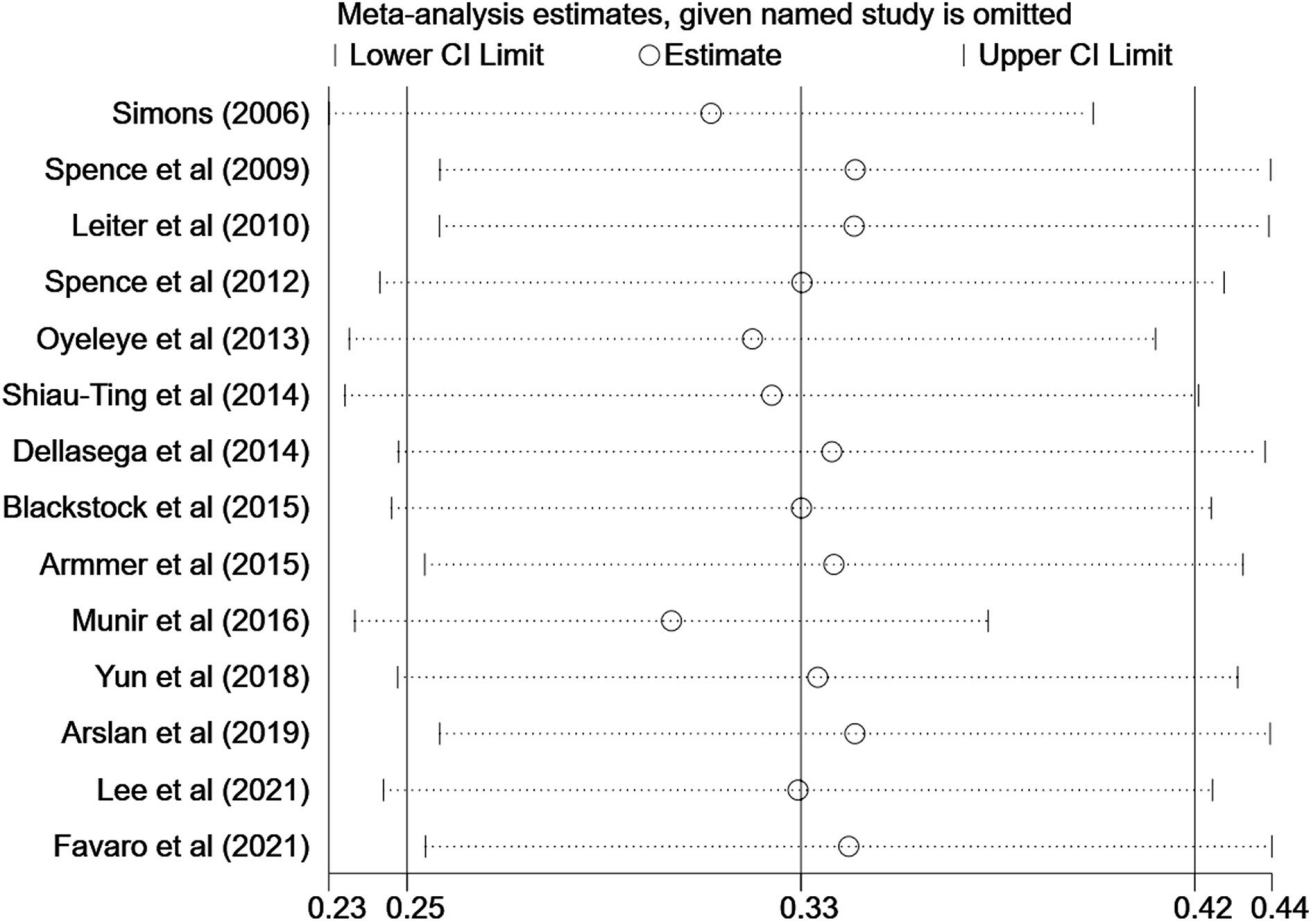
Sensitivity analysis estimating heterogeneity.

**Figure 4:**
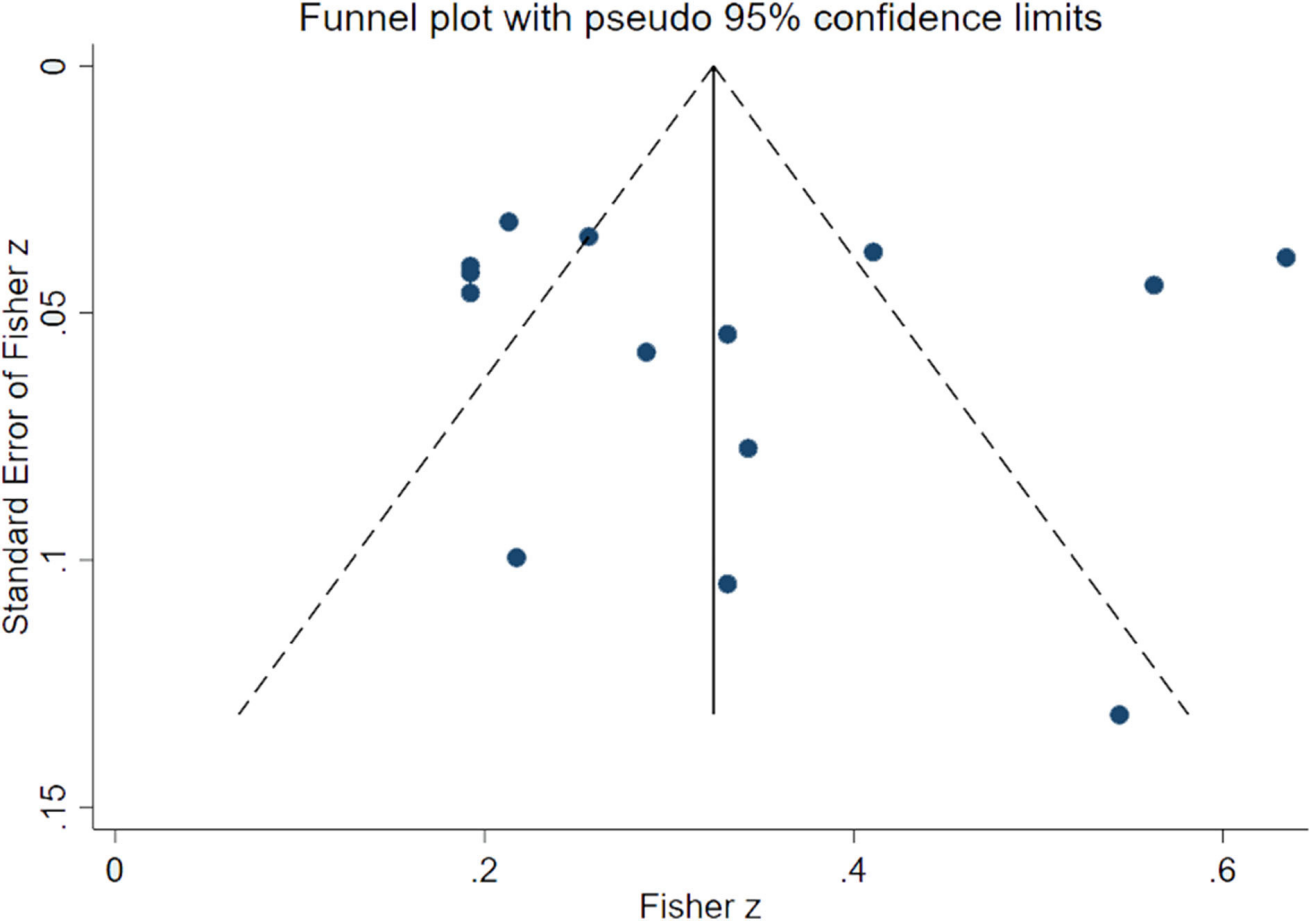
Funnel plots estimating small sample bias.

**Figure 5:**
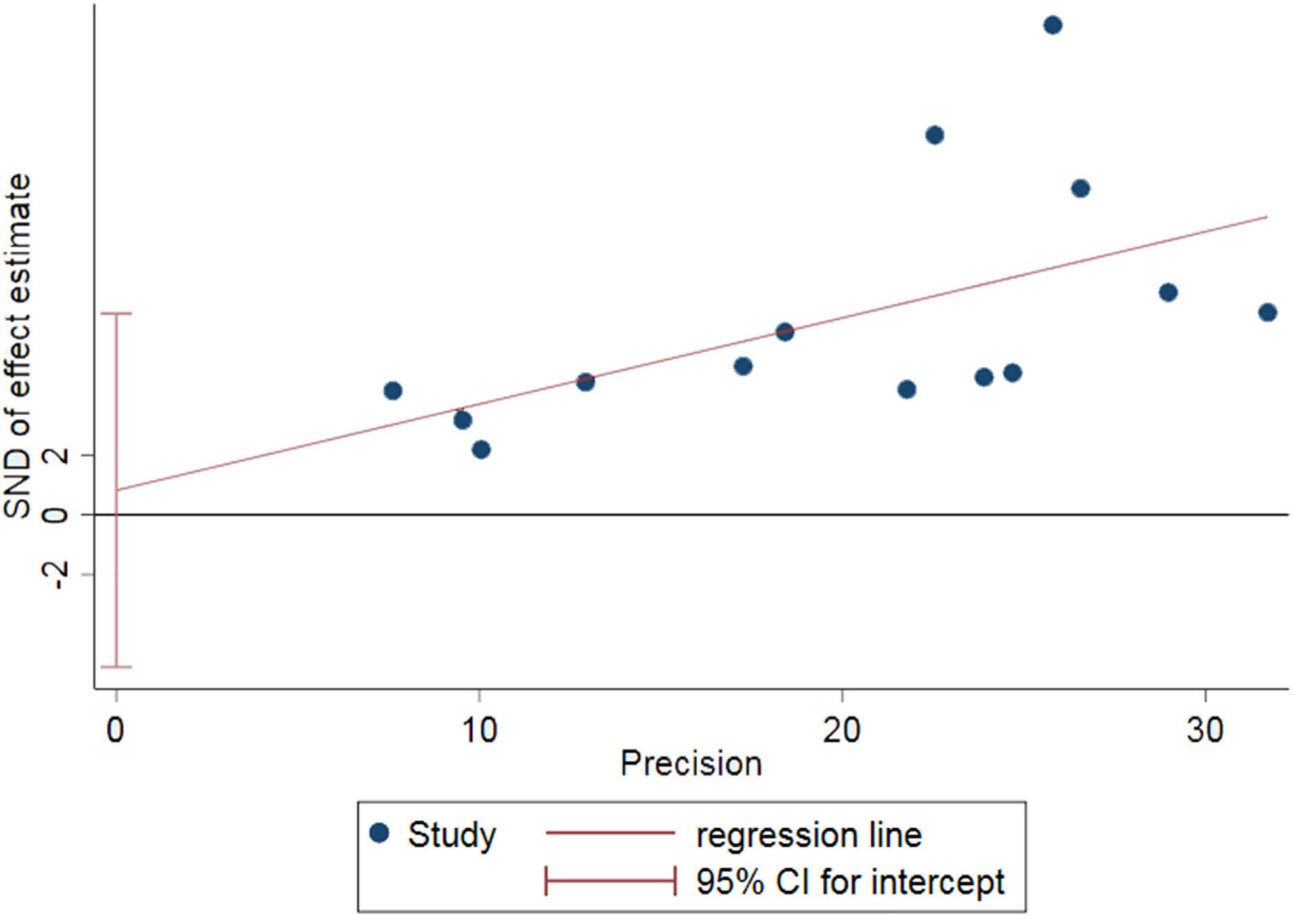
Egger's test estimating publication bias.

Although the quality of the two studies was assessed as high risk, both sensitivity analysis and subgroup analysis showed no significance, indicating that the risk of bias was not related to study quality, so two low-quality studies were still included in the meta-analysis. Sensitivity analysis was conducted on the included 14 studies, and it was found that the results were unchanged when each study was excluded serially (Figure 3). It indicated that the results of this meta-analysis were stable. Funnel plots and Egger's test indicated that no significant evidence of publication bias was found in the 14 studies (Egger bias = 0.82, 95% CI: (−5.09, 6.73), *P* = 0.767) (Figures 4, 5).

## Discussion

This systematic review and meta-analysis quantitatively assessed the association between horizontal violence and turnover intention in nurses. As far as we know, the review included 14 studies involving 6,472 nurses and was the first quantitative assessment of the association between horizontal violence and turnover intention. The results of the random-effects meta-analysis procedure show that there is a positive correlation between horizontal violence and turnover intention in nurses.

Subgroup analysis of the geographic region, sample size, horizontal violence measurement tools, and quality showed that only horizontal violence measurement tools might be the source of heterogeneity (*P* = 0.001). NAQ-R (Fisher *z* = 0.45; *Z* = 6.901, *P* < 0.001) showed a stronger correlation between horizontal violence and turnover intention than WIS (Fisher *z* = 0.19; *Z* = 7.822, *P* < 0.001) and Others (Fisher *z* = 0.27; *Z* = 8.178, *P* < 0.001). The results showed that horizontal violence associated with turnover intention in Asia (Fisher *z* = 0.38; *Z* = 4.406, *P* < 0.001) was stronger than that in America (Fisher *z* = 0.30; *Z* = 6.510, *P* < 0.001), but there was no statistical significance between groups (*P* = 0.463). Further divided by different countries (USA, Canada, and Korea) into subgroups, heterogeneity was found among different countries (*P* < 0.001): USA (Fisher *z* = 0.39; *Z* = 3.792, *P* < 0.001), followed by Korea (Fisher *z* = 0.31; *Z* = 6.631, *P* < 0.001). The Canada correlation was considered almost irrelevant (Fisher *z* = 0.23; *Z* = 8.728, *P* < 0.001). It suggested that the correlation between horizontal violence and turnover intention was different in countries, which might be related to the cultural environment. Influenced by cultural differences, individuals may react differently to violent acts (13). As suggested by Sorge et al. (44), the extent to which members of a specific culture can control their desires and impulses is one of the influential dimensions used to classify that culture. Workplace incivility tends to be higher in “indulgent” cultures (such as the USA), while “restrained” cultures (such as Mediterranean countries) have weaker impulse control (44). Lutgen et al. (45) noted that in individualistic cultures, such as the USA, the individual may feel more threatened by bullying and more challenged by bullying events since they may perceive bullying as an attempt to weaken their competitive strength. In the context of collectivism, for example, in Asian countries, such as China and Japan, harmony and group norms are more valuable, which may lead nurses to choose to avoid or even get used to their negative emotions after experiencing bullying and return to their previous level of well-being after a period of time (46). It should be noted that the number of studies included in this meta-analysis was insufficient, and there was insufficient evidence for a pooled analysis when conducting subgroup analysis. Therefore, it is suggested that researchers should conduct more comparative studies on cultural differences in the future. In addition, the correlation measured by the NAQ-R measurement tool was significantly higher than that of the other two groups (Fisher *z* = 0.45; *Z* = 6.901, *P* < 0.001). WIS (Fisher *z* = 0.19; *Z* = 7.822, *P* < 0.001) and Others (Fisher *z* = 0.27; *Z* = 8.178, *P* < 0.001) showed almost no correlation between horizontal violence and turnover intention. This may be related to NAQ-R's sensitivity to horizontal violence. Currently, NAQ-R is the main measurement tool used to measure horizontal violence. Among the 14 studies included in this review, it can also be found that there are six studies (32–34, 38, 40, 41) used NAQ-R, one of which is the third edition of NAQ (33), and three of the five studies after 2015 have used this measurement tool. It is suggested that researchers conduct controlled trials to further explore the differences in horizontal violence measured with different measurement tools in the future.

The advantage of this systematic review and meta-analysis include: previous studies have inconsistent conclusions on the correlation between horizontal violence and turnover intention in nurses, while this review, including 6,472 nurses, has a large sample size and a relatively firm conclusion. Through sensitivity analysis and heterogeneity test, it was finally confirmed that the result was stable. Therefore, it can provide reference for future nursing managers to pay attention to horizontal violence, implement intervention measures and reduce the turnover intention of nurses. In addition, a meta-analysis showed a pooled *r-*value of 0.32 (95% CI: 0.29–0.34; *P* < 0.001), and there was a low-positive correlation between nurses' horizontal violence and turnover intention. Horizontal violence has been shown to be one of the reasons nurses leave their jobs. It is suggested that nurse managers should not only pay attention to the improvement of nurses' professional ability and the prevention of nurse-patient conflict, but also pay attention to the impact of negative behavior among colleagues on nurses, and explore strategies to improve horizontal violence.

This meta-analysis has the following limitations. First, the number of studies included in this meta-analysis is small, and the number of subgroup analysis studies is insufficient. For example, the studies are limited to Asia and America, there are no studies from other regions due to some reasons, which limits the representativeness of the results. So, it is recommended that studies on the correlation between horizontal violence and turnover intention be carried out in more regions and that meta-analysis results must be updated to better represent global levels. Second, the outcomes caused by horizontal violence are not limited to turnover intention. Although most studies suggest that a higher turnover rate is related to horizontal violence, this study suggests a low correlation. In recent years, with the development of structural equation modeling, more and more studies have focused on the mechanism between them. Therefore, it is suggested to explore the relationship between horizontal violence and other outcomes in the future and explore variables that may play a mediating or moderating effect between horizontal violence and turnover intention. Finally, we tried our best to have a rigorous attitude for screening and analysis of interpretation of the study, but the research mainly was a cross-sectional design. Horizontal violence and turnover intention at the same time measurement may lead to the possibility of a spurious correlation. So, there is a need for more longitudinal studies, which can further explain the correlation between horizontal violence and turnover intention.

## Conclusion

This meta-analysis suggests that horizontal violence has a low-negative correlation with turnover intention in nurses. Nurses who have experienced horizontal violence tend to be more likely to leave their jobs than those who have not experienced horizontal violence. Awareness of this correlation may prompt nursing managers to pay more attention to horizontal violence, care about how nurses get along with colleagues, and strive to create a good working atmosphere, thereby reducing nurse turnover.

## Data availability statement

The original contributions presented in the study are included in the article/Supplementary material, further inquiries can be directed to the corresponding author/s.

## Author contributions

YZ and JL searched and checked the databases according to the inclusion and exclusion criteria, extracted the data, and assessed their quality. YZ analyzed the data and wrote the draft of the paper. RY and JC gave advice on meta-analysis methodology and revised the paper. LM is the guarantor of this work and had full access to all the data in the study and takes responsibility for its integrity and the accuracy of the data analysis. All authors contributed to reviewing, read, and approved the final manuscript.

## Funding

The authors received financial support from the National Natural Science Foundation of China (Grant No. 71874117).

## Conflict of interest

The authors declare that the research was conducted in the absence of any commercial or financial relationships that could be construed as a potential conflict of interest.

## Publisher's note

All claims expressed in this article are solely those of the authors and do not necessarily represent those of their affiliated organizations, or those of the publisher, the editors and the reviewers. Any product that may be evaluated in this article, or claim that may be made by its manufacturer, is not guaranteed or endorsed by the publisher.
