# Supplementary material for: Association between horizontal violence and turnover intention in nurses: A systematic review and meta-analysis
Source: Front Public Health. 2022 Oct 6;10:964629. doi: 10.3389/fpubh.2022.964629 (PMC9583538; doi:10.3389/fpubh.2022.964629)
Supplement: Supplementary file 1 [file Data_Sheet_1.docx]

**Supplementary file 1:** Search strategy

PubMed

| Search number | Query |
| --- | --- |
| 5 | ((nurses[MeSH Terms]) OR ((nurses[Title/Abstract]) OR (Nurse[Title/Abstract]) OR (Personnel, Nursing[Title/Abstract]) OR (Nursing Personnel[Title/Abstract]) OR (Registered Nurses[Title/Abstract]) OR (Nurse, Registered[Title/Abstract]) OR (Nurses, Registered[Title/Abstract]) OR (Registered Nurse[Title/Abstract]))) AND ((lateral violence[Title/Abstract]) OR (horizontal violence[Title/Abstract]) OR (horizontal hostility[Title/Abstract]) OR (bullying[Title/Abstract]) OR (workplace incivility[Title/Abstract])) |
| 4 | (lateral violence[Title/Abstract]) OR (horizontal violence[Title/Abstract]) OR (horizontal hostility[Title/Abstract]) OR (bullying[Title/Abstract]) OR (workplace incivility[Title/Abstract]) |
| 3 | (nurses[MeSH Terms]) OR ((nurses[Title/Abstract]) OR (Nurse[Title/Abstract]) OR (Personnel, Nursing[Title/Abstract]) OR (Nursing Personnel[Title/Abstract]) OR (Registered Nurses[Title/Abstract]) OR (Nurse, Registered[Title/Abstract]) OR (Nurses, Registered[Title/Abstract]) OR (Registered Nurse[Title/Abstract])) |
| 2 | (nurses[Title/Abstract]) OR (Nurse[Title/Abstract]) OR (Personnel, Nursing[Title/Abstract]) OR (Nursing Personnel[Title/Abstract]) OR (Registered Nurses[Title/Abstract]) OR (Nurse, Registered[Title/Abstract]) OR (Nurses, Registered[Title/Abstract]) OR (Registered Nurse[Title/Abstract]) |
| 1 | nurses[MeSH Terms] |

Embase

| No. | Query |
| --- | --- |
| #5 | #2 AND #4 |
| #4 | #1 AND #3 |
| #3 | nurses':ab,ti OR 'nurse':ab,ti OR 'personnel, nursing':ab,ti OR 'nursing personnel':ab,ti OR 'registered nurses':ab,ti OR 'nurse, registered':ab,ti OR 'nurses, registered':ab,ti OR 'registered nurse':ab,ti |
| #2 | 'lateral violence':ab,ti OR 'horizontal violence':ab,ti OR 'horizontal hostility':ab,ti OR 'bullying':ab,ti OR 'workplace incivility':ab,ti |
| #1 | 'nurse'/exp |

Cochrane

| ID | Search |
| --- | --- |
| #1 | MeSH descriptor: [Nurses] explode all trees |
| #2 | (nurses):ti,ab,kw OR (Nurse):ti,ab,kw OR (Personnel, Nursing):ti,ab,kw OR (Nursing Personnel):ti,ab,kw OR (Registered Nurses):ti,ab,kw OR (Nurse, Registered):ti,ab,kw OR (Nurses, Registered):ti,ab,kw OR (Registered Nurse):ti,ab,kw |
| #3 | #1 OR #2 |
| #4 | (lateral violence):ti,ab,kw OR (horizontal violence):ti,ab,kw OR (horizontal hostility):ti,ab,kw OR (bullying):ti,ab,kw OR (workplace incivility):ti,ab,kw |
| #5 | #3 AND #4 |

CINAHL

| # | Query |
| --- | --- |
| S6 | (S1 AND S2) AND (S4 OR S5) |
| S5 | S1 AND S2 |
| S4 | S1 AND S2 |
| S3 | S1 AND S2 |
| S2 | TI lateral violence OR AB lateral violence OR TI horizontal violence OR AB horizontal violence OR TI horizontal hostility OR AB horizontal hostility OR TI bullying OR AB bullying OR TI workplace incivility OR AB workplace incivility |
| S1 | TI nurses OR AB nurses OR TI Nurse OR AB Nurse OR TI Personnel, Nursing OR AB Personnel, Nursing OR TI Nursing Personnel OR AB Nursing Personnel OR TI Registered Nurses OR AB Registered Nurses OR TI Nurse, Registered OR AB Nurse, Registered OR TI Nurses, Registered OR AB Nurses, Registered OR TI Registered Nurse OR AB Registered Nurse |

CNKI / Wanfang / SinoMed:

(lateral violence OR horizontal violence OR horizontal hostility OR bullying OR workplace incivility) And nurse.
