# Supplementary material for: Association between horizontal violence and turnover intention in nurses: A systematic review and meta-analysis
Source: Front Public Health. 2022 Oct 6;10:964629. doi: 10.3389/fpubh.2022.964629 (PMC9583538; doi:10.3389/fpubh.2022.964629)
Supplement: Supplementary file 2 [file Data_Sheet_2.docx]

**Supplementary 2 Subgroup analysis of the pooled results of the correction between horizontal violence and turnover intention**


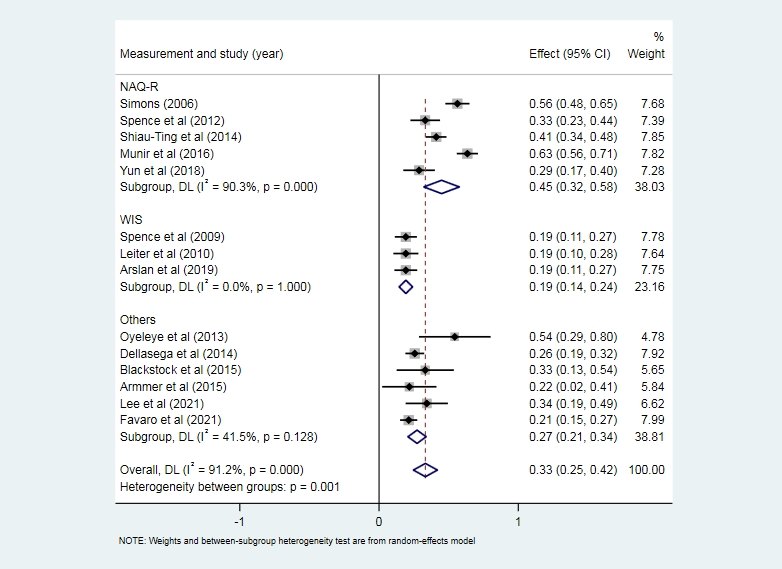


Subgroup analysis of measurement tool of horizontal violence and turnover intention in pooled Fisher z analysis.


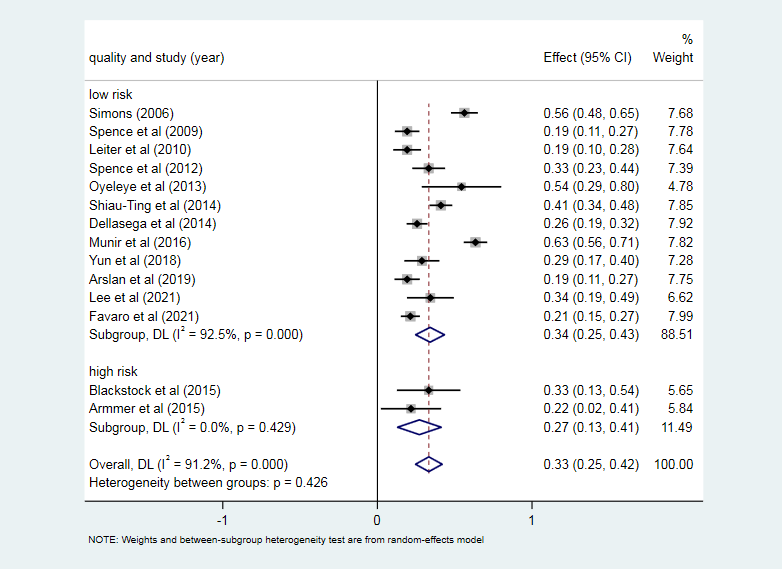


Subgroup analysis of quality of horizontal violence and turnover intention in pooled Fisher z analysis.


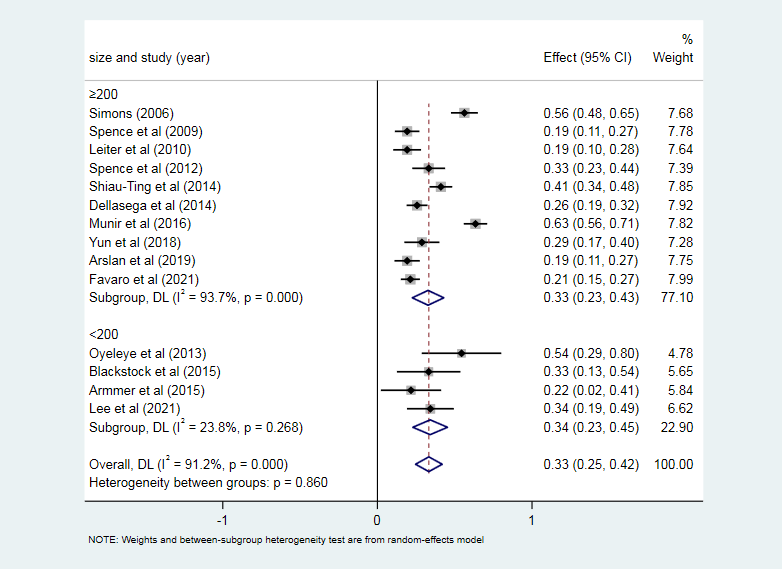


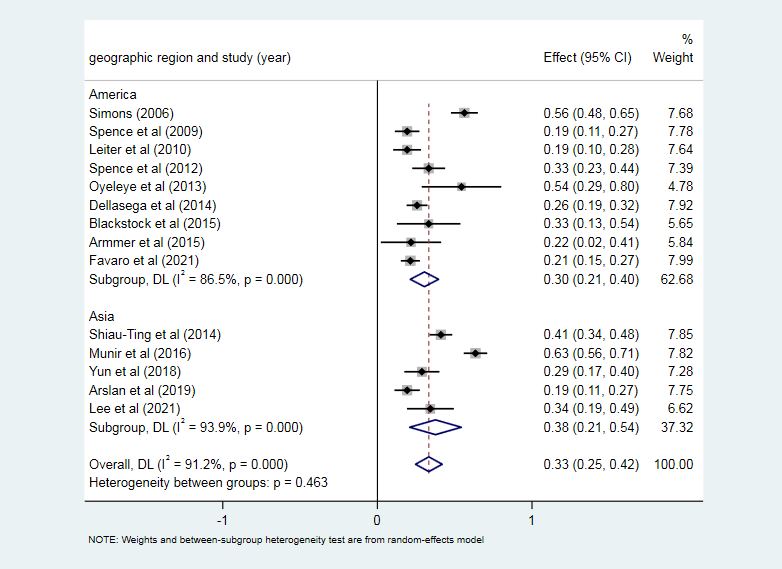


Subgroup analysis of sample size of horizontal violence and turnover intention in pooled Fisher z analysis.


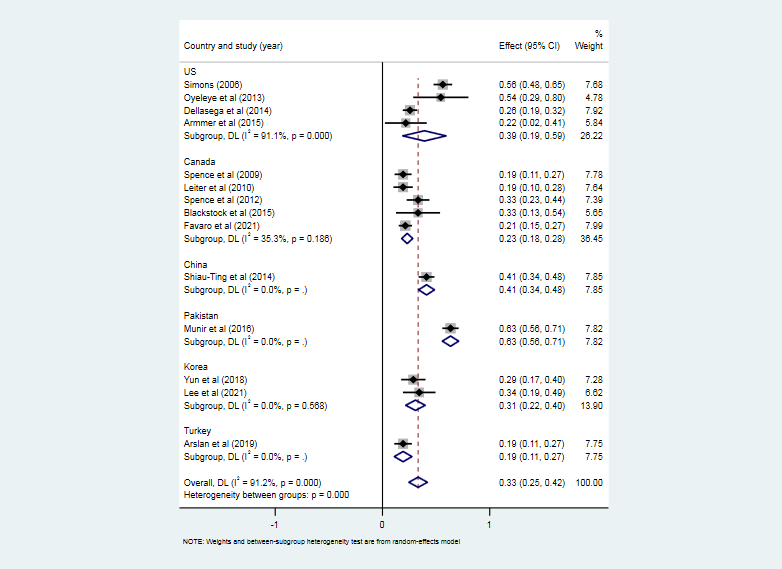


Subgroup analysis of country of horizontal violence and turnover intention in pooled Fisher z analysis.
